# Comparison of MLC positioning deviations using log files and establishment of specific assessment parameters for different accelerators with IMRT and VMAT

**DOI:** 10.1186/s13014-022-02097-0

**Published:** 2022-07-16

**Authors:** Xiutong Lin, Tao Sun, Xiao Liu, Guifang Zhang, Yong Yin

**Affiliations:** Department of Radiation Physics and Technology, Shandong Cancer Hospital and Institute, Shandong First Medical University and Shandong Academy of Medical Sciences, 440 Jiyan Road, Jinan, 250117 Shandong China

**Keywords:** MLC positioning error, Trajectory log file, Dynalog, IMRT, VMAT, Trilogy, TrueBeam, Halcyon

## Abstract

**Background and purpose:**

The study evaluated the differences in leaf positioning deviations by the log files of three advanced accelerators with two delivery techniques, and established specific assessment parameters of leaf positioning deviations for different types of accelerators.

**Methods:**

A total of 420 treatment plans with 5 consecutive treatment log files were collected from the Trilogy, TrueBeam and Halcyon accelerators. Millennium MLC was equipped on the Trilogy and TrueBeam accelerators. A jawless design and dual-layer MLC were adopted on the Halcyon accelerator. 70 IMRT and 70 VMAT plans were selected randomly on each accelerator. The treatment sites of all plans included head and neck, chest, breast, pelvis and other sites. The parsing tasks for 2100 log files were proceeded by SunCheck software from Sun Nuclear Corporation. The maximum leaf root mean square (RMS) errors, 95th percentile errors and percentages of different leaf positioning errors were statistically analyzed. The correlations between these evaluation parameters and accelerator performance parameters (maximum leaf speed, mean leaf speed, gantry and arc angle) were analyzed.

**Results:**

The average maximum leaf RMS errors of the Trilogy in the IMRT and VMAT plans were 0.44 ± 0.09 mm and 0.79 ± 0.07 mm, respectively, which were higher than the TrueBeam's 0.03 ± 0.01 mm, 0.03 ± 0.01 mm and the Halcyon's 0.05 ± 0.01 mm, 0.07 ± 0.01 mm. Similar data results were shown in the 95th percentile error. The maximum leaf RMS errors were strongly correlated with the 95th percentile errors (Pearson index > 0.5). The leaf positioning deviations in VMAT were higher than those in IMRT for all accelerators. In TrueBeam and Halcyon, leaf position errors above 1 mm were not found in IMRT and VMAT plans. The main influencing factor of leaf positioning deviation was the leaf speed, which has no strong correlation with gantry and arc angles.

**Conclusions:**

Compared with the quality assurance guidelines, the MLC positioning deviations tolerances of the three accelerators should be tightened. For both IMRT and VMAT techniques, the 95th percentile error and the maximum RMS error are suggested to be tightened to 1.5 and 1 mm respectively for the Trilogy accelerator. In TrueBeam and Halcyon accelerators, the 95th percentile error and maximum RMS error of 1 and 0.5 mm, respectively, are considered appropriate.

**Supplementary Information:**

The online version contains supplementary material available at 10.1186/s13014-022-02097-0.

## Introduction

The multileaf collimator (MLC) is one of the key components of the accelerator, and its invention has become an important part of accurate radiotherapy. With the development, the MLCs were constantly changing in different types of accelerators, such as Varian's C-series accelerators, and later updated T-series and O-series accelerators. Intensity modulated radiation therapy (IMRT) and volumetric modulated arc therapy (VMAT) techniques can achieve more conformal dose distribution with the dynamic MLCs. Previous studies have shown that the dose distribution could be directly affected by MLC positioning deviations [1, 2], so the use of specific MLC assurance procedure is recommended for the accelerators providing IMRT or VMAT delivery [3].

The MLC log files record the data of the MLCs during the dose delivery, containing MLC leaf positioning and speed. These log files can help to find MLC positioning errors or be used for specific quality assurance (QA) [4–6]. Previous studies [7–10] have verified the accuracy of log file data with film, diode array, and electronic portal image device (EPID), so log files can be an effective tool for IMRT and VMAT delivery verification.

Traditional QA guidelines have inconsistent specifications for MLC positioning tolerances. The TG-51 recommends a tolerance of 0.5 mm for static MLC leaf position accuracy, and the values of the maximum leaf root mean square (RMS) and 95th percentile leaf positioning error were not proposed [11]. In TG-142 and the latest TG-198, the tolerance of MLC leaf position accuracy is ± 1 mm, and the RMS and 95th percentile errors should be < 0.35 cm [12, 13]. The above guidelines recommended annual assessments for the both tests. The recommended reference values in these guidelines may lag due to the changes in the MLC structures of accelerators and the continuous improvement of equipment accuracy.

Although there have been many studies on MLC log files in the past, no studies have established more detailed tolerances of MLC positioning deviations relative to the guidelines based on different types of accelerators (especially more advanced accelerators) and different radiotherapy techniques.

This study is based on Varian's Trilogy (C-series), TrueBeam (T- series), and Halcyon (O- series) accelerators. We reviewed MLC log files of the three accelerators with IMRT and VMAT techniques. These log files are not the standard test files used for annual QA, but the actual treatment records. By comparing the log files, the differences of MLC position errors between different accelerators and techniques were analyzed to determine the variability. Finally, in order to better evaluate the MLC performance of different accelerators and provide help for the prospective detection and avoidance of MLC position errors, we established individualized action thresholds and evaluation values of MLC position errors for different accelerators.

## Methods and materials

### Equipment

Three Varian accelerators (Varian Medical Systems, Palo Alto, CA, USA) were involved in this study. The mechanical characteristics of the three accelerators are described below:

A. Trilogy accelerator: This accelerator and previous Varian accelerators are collectively referred to as the "C-series", but it is more advanced than the previous series and can implement VMAT technique. It is equipped with a Millennium MLC, and the projection width at the isocenter are 5 and 10 mm in the middle 80 leaves and the outer 40 leaves respectively. For the Millennium MLC, the thickness of the leaf made of tungsten alloy is 6.5 cm. The maximum leaf speed is 2.5 cm/s. A passive MLC controller is used. The version of the Trilogy accelerator is model SN: H296054, and the version number of the MLC controller is 8.1.10.1. Only flattening filter (FF) beams were available on the Trilogy accelerator in our hospital.
B. TrueBeam accelerator: This accelerator is one of the new series of digital representatives, known as the “T-series”. The Millennium MLC is also used in this accelerator. Although the mechanical parameters of the MLCs in TrueBeam are the same as those in Trilogy. The accelerator has a more perfect integrated digital control system and uses an active MLC controller. The full system version is 02.05.3001. In this study, all treatment plans on the Truebeam were based on FF mode.
C. Halcyon accelerator: It is the latest accelerator developed by Varian company, known as the “O-series” because of its O-shell setup. The design of the MLCs is different from the structure of the traditional MLC. Jaw is removed and double-layer MLCs are used instead. There are 29 pairs and 28 pairs leaves at the proximal bank and distal bank respectively. The projection width at the isocenter is 1 cm, and the maximum leaf speed is 5 cm/s. The thickness of the leaf is 7.7 cm, and is made of tungsten alloy. The MLC controller adopted is active, and the full system version of Halcyon is 2.0.100.3. Halcyon offers a single 6 MV flattening-filter-free (FFF) X-ray.

On each accelerator, 70 IMRT and 70 VMAT plans were randomly selected. IMRT and VMAT plans were designed in the Varian Eclipse version 15.5 treatment planning system (Varian Medical Systems, Palo Alto, CA, USA). 6 MV X-ray was used in all plans. The numbers of fields and arcs were determined by the physicists according to the location of the patient's tumor and the difficulty of the plan. When selecting the plan, we did not require the number of fields. The dose rates were set at 400 MU/min in the IMRT plans. In the VMAT plans, the maximum dose rates were 600 MU/min in Trilogy and TrueBeam plans and 800 MU /min in the Halcyon plans respectively. Sliding window technique was used in the IMRT plans which means the MLC moves continuously when beam is on. In the VMAT plans, when beam is on, the MLC position, leaf speed, gantry, and dose rate may all change.

The treatment plans of the three machines were randomly selected, and the treatment sites included the head and neck, chest (esophagus or lung), breast, pelvic, and others. Although the numbers of categories of treatment site were different (for example, Halcyon has the most pelvic plans and the least breast plans), the planning field sizes of the same treatment site were similar in the three machines, that is, the planning complexity of the same site was similar. Each machine contained more than five categories of treatment sites. The planning complexity of different sites was not compared. The related detailed parameters of the 420 plans for the three machines are shown in Additional file 1: Table 1.

### MLC log files

MLC log files are created by the MLC controller. The recording modes of log files have changed with the development of equipment. Before Varian's T-series accelerators, the C-series accelerators (such as Trilogy, EX, iX) adopted passive log file recording methods, and their files were named Dynalogs. The passive MLC controller has a 50 ms communication delay, which means that the leaves are delayed 50 ms with regard to the planned position during treatment delivery. After each field delivery, the MLC controller automatically saves two dynamic log files, one for each bank. The log files record the treatment delivery parameters every 50 ms, including the MUs, collimator and gantry angle, expected (planned) MLC positions, and the actual MLC positions recorded by the encoder connected to the motor on each leaf [14]. Through these log files, the positioning errors of MLCs can be calculated.

Starting from Varian's T-series accelerator, such as TrueBeam, VitalBeam and Halcyon, the log file recording modes are active. In these accelerators, the leaves will not be delayed to the planned position due to the active MLC controller. Therefore, there is no delay effect in MLC positioning. The log files are named Trajectory logs. Trajectory logs are binary files recording planned and actual MLCs positions at a sampling rate of 50 Hz (20 ms) [15]. Because of the active MLC controller design, the leaves will not be delayed in the planned position. Therefore, compared to Trilogy accelerators, TrueBeam and Halcyon accelerators have no delay effect on MLC positioning [16].

During the machine is running, it will monitor the accuracy of the dynamic MLC, and the device will set a “dynamic threshold” to prevent excessive errors. For Trilogy, factory defaults are 2 mm for sliding window IMRT and 5 mm for VMAT. The MLC control system samples MLC position every 50 ms and compares the leaf actual and planned position. If the leaf position deviation is out of tolerance, a beam hold takes place to allow the leaf to catch up. If the leaf can't catch up in 60 feedback cycles (approximate 3 s) then a “dynamic position” interlock will take place [17]. For TrueBeam and Halcyon, the threshold set in the Eclipse treatment planning system is 2 mm.

### Data analysis

Machine QA function in Suncheck version 3.1 software (Sun Nuclear Corporation, Melbourne, Florida, USA) was used to establish MLC positioning and leaf speed analysis projects for the three accelerators, and one analysis project was generated for each patient. Real treatment log files of 70 IMRT and other 70 VMAT plans were collected from each accelerator. Log files of 2100 treatment fractions from five consecutive deliveries on 420 plans were analyzed, including MLC position and leaf speed.

The analysis parameters of leaf position errors were maximum leaf RMS error, 95th percentile error, the number of failed leaves and the percentage of different leaf positioning errors. The first two analysis parameters are recommended in the TG-142 and TG-198 report, and the report believes that these two parameters are helpful for analyzing the state of the MLC performance [12, 13]. The maximum leaf RMS error is a single value, which is the maximum RMS error of all leaves in bank A and bank B. It takes into account the error of each leaf during the entire dose delivery, regardless of the direction of the positioning error. The 95th percentile error is a single value extracted from the list of leaf position errors. The threshold value of leaf position error was set to ± 1 mm due to the existence of two error directions of the leaf position. Failed leaves referred to the leaves with the positioning error exceeding the threshold in any direction. The percentages of the number of leaves with different position error values of 0, ± 0.05 mm, ± 0.5 mm, and ± 1 mm were counted. The analyzed maximum and mean leaf speed were the maximum and mean speed of the single leaf, and the single leaf speed was calculated independent of the beam state.

### Statistical analysis

All data were statistically analyzed using the Origin v10.5 software (OriginLab Corporation, Northampton, Massachusetts, USA). The data of different accelerators and dose delivery techniques were grouped, and the non-parametric test was used to analyze the differences of the leaf position deviations among the groups. The differences were considered statistically significant when *p* < 0.05. Correlation analysis was conducted among the analysis parameters of leaf position deviations. The correlations between the evaluation parameters of leaf positioning errors and the mechanical parameters (maximum and mean leaf speed, gantry or arc angles) were analyzed. Pearson correlation coefficient was adopted for correlation analysis, and the correlation coefficients between ± (0.5, 1) were considered to be strongly correlated.

## Results

### Comparisons of bank A and bank B for the three accelerators

**Fig. 1:**
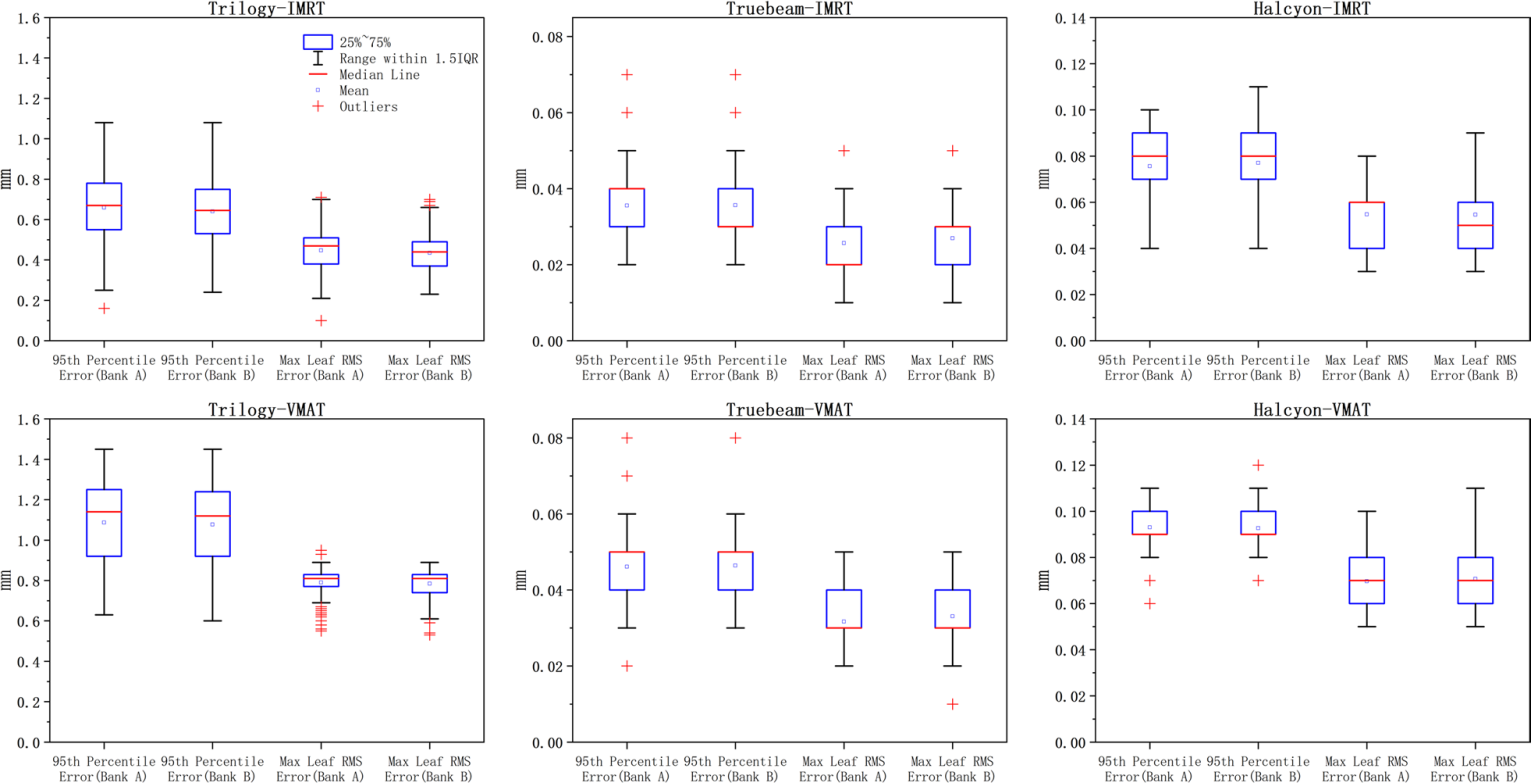
Box plot of max leaf RMS and 95th percentile error values in the three accelerators with IMRT and VMAT techniques. The edges of the box represent the 75th (q3) and 25th (q1) percentile values and the middle mark represents the median. The upper and the lower lines represent the largest and the smallest non-outlier values. Data points higher than q3 + 1.5 or lower than q1-1.5 are considered and painted in red as outliers

**Fig. 2:**
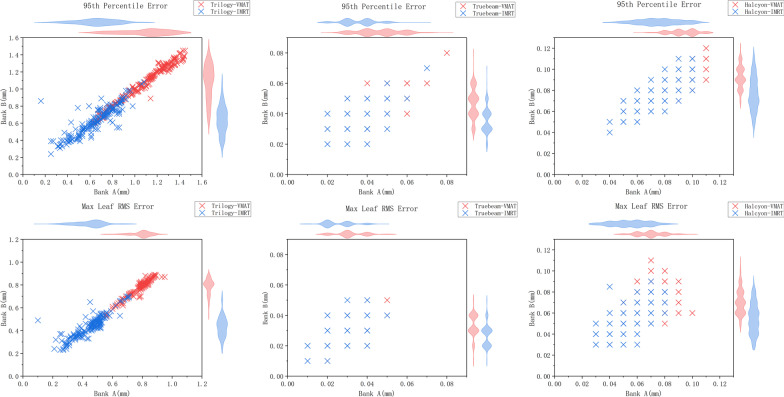
Correlation analysis of bank A and bank B in the maximum leaf RMS and 95th percentile errors of the three accelerators with IMRT and VMAT. The horizontal axis of each graph is MLC in the direction of bank A, and the vertical axis is in the direction of bank B. The blue cross scatter chart in the center represents the MLC evaluation parameter (95th or RMS) when IMRT is performed, while red represents VMAT. The violin pictures corresponding to the horizontal and vertical axes are displayed at the top and right of each figure, and the thicker the part, the higher the frequency of the corresponding value

The box plots of the maximum leaf RMS and 95th percentile errors for the three accelerators with IMRT and VMAT techniques are shown in Fig. 1. Correlations were observed between bank A and bank B in the two leaf-position-error evaluation parameters of all accelerators and techniques, and the Pearson correlation coefficients were all greater than 0.5, as shown in Fig. 2. Compared to IMRT, VMAT increased the maximum leaf RMS and 95th percentile errors in all accelerators.

### Comparison between the three accelerators

For the IMRT technique, the mean values of maximum leaf RMS error and 95th percentile error were 0.44 ± 0.1 mm, 0.65 ± 0.17 mm in Trilogy, 0.03 ± 0.01 mm, 0.04 ± 0.01 mm in TrueBeam, and 0.05 ± 0.01 mm, 0.08 ± 0.02 mm in Halcyon respectively. For the VMAT technique, the mean values of maximum leaf RMS error and 95th percentile error were 0.79 ± 0.07 mm, 1.08 ± 0.21 mm in Trilogy, 0.03 ± 0.01 mm, 0.05 ± 0.01 mm in TrueBeam, and 0.07 ± 0.01 mm, 0.09 ± 0.01 mm in Halcyon, respectively. The data are shown in Table 1. The maximum leaf RMS and 95th percentile errors in Trilogy were significantly higher than in the other two accelerators. Halcyon significantly increased the values of the two parameters compared to TrueBeam. The differences in the pairwise comparison of the three accelerators were statistically significant (*p* < 0.05). Additional file 1: Fig. 1 shows the comparison of maximum leaf RMS error and 95th percentile error among the three accelerators with IMRT and VMAT technique.

**Table 1:** Evaluation parameters of leaf positioning errors for IMRT and VMAT plans on the three accelerators

		95th error (mm)	Max RMS error(mm)	Max leaf speed (mm/s)	Mean leaf speed (mm/s)	Fail leaves number
Trilogy	IMRT	0.65 ± 0.17	0.44 ± 0.10	22.41 ± 2.21	3.50 ± 1.14	4.88 ± 4.40
	VMAT	1.08 ± 0.21	0.79 ± 0.07	35.40 ± 2.93	7.88 ± 0.64	21.07 ± 7.01
TrueBeam	IMRT	0.04 ± 0.01	0.03 ± 0.01	22.58 ± 1.95	1.64 ± 0.91	0
	VMAT	0.05 ± 0.01	0.03 ± 0.01	23.23 ± 2.60	4.14 ± 1.65	0
Halcyon	IMRT	0.08 ± 0.02	0.05 ± 0.01	55.72 ± 1.29	3.00 ± 1.19	0
	VMAT	0.09 ± 0.01	0.07 ± 0.01	58.86 ± 1.06	11.92 ± 3.29	0

*IMRT* intensity-modulated radiation therapy, *VMAT* volumetric-modulated arc therapy

### Separate analysis for each accelerator

#### Trilogy

**Fig. 3:**
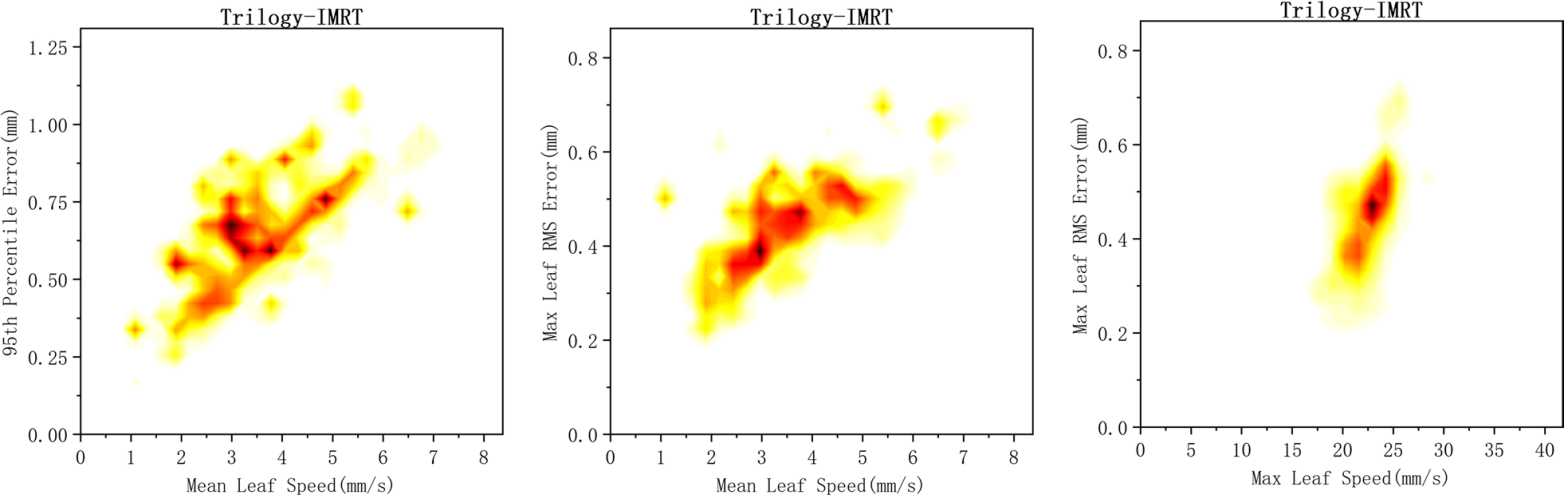
The density plots of correlation between the maximum leaf RMS and the 95th percentile error and leaf speed in Trilogy with IMRT technique. Data densities are indicated by color changes. Yellow represents low density, red represents high density, and black represents the highest density

In IMRT plans, the maximum leaf RMS and 95th percentile error showed strong correlation with the mean leaf speed, with R values of 0.70 (*p* = 0.00) and 0.67 (*p* = 0.00), respectively. The maximum leaf RMS error also showed a correlation with the maximum leaf speed (R = 0.65, *p* = 0.00). There was no correlation between the 95th percentile error and the maximum leaf speed (R = 0.29, *p* = 0.00). Figure 3 shows the density plot which displayed the maximum leaf RMS and 95th percentile error as a function of the maximum and mean leaf speed in IMRT plans.

In VMAT plans, a strong correlation was observed between the maximum leaf RMS error and the maximum leaf speed (R = 0.84, *p* < 0.05) and mean leaf speed (R = 0.72, *p* < 0.05). The 95th percentile error was also correlated with the maximum leaf speed (R = 0.84, *p* < 0.05) and mean leaf speed (R = 0.61, *p* < 0.05). The density plots in Additional file 1: Fig. 2 shows the maximum leaf RMS and 95th percentile error as a function of the maximum and mean leaf speed in VMAT plans.

Through the analysis of the number of failed leaves with a position error of more than 1 mm, it was found that the number of failed leaves exceeding 1 mm was correlated with the 95th percentile and maximum leaf RMS errors both in IMRT and VMAT plans (R > 0.5, *p* < 0.05). The number of failed leaves in VMAT was also correlated with the mean leaf speed (R = 0.55, P = 0.00), and the number of failed leaves increased with the increase of leaf speed. The data are shown in Additional file 1: Fig. 3. There was no correlation between the number of failed leaves and the maximum leaf speed, and the correlation coefficients were 0.36 and 0.48 in IMRT and VMAT, respectively. Additional file 1: Table 2 showed the specific values of the correlation coefficients among the evaluation parameters.

#### TrueBeam

In IMRT plans, the 95th percentile error was correlated with maximum and mean leaf speed (R = 0.52 and 0.60, respectively). No correlation was observed between the maximum leaf RMS error and the maximum and mean leaf speed (R = 0.41 and 0.47, respectively).

In VMAT plans, both the maximum leaf RMS and the 95th percentile error were correlated with the mean leaf speed (R = 0.57 and 0.70, p < 0.05). No correlation was observed between the maximum leaf RMS and 95th percentile error and the maximum leaf speed (R = 0.21 and 0.27, respectively). Additional file 1: Fig. 4 shows the correlation between the maximum leaf RMS and 95th percentile error and leaf speed in TrueBeam.

**Fig. 4:**
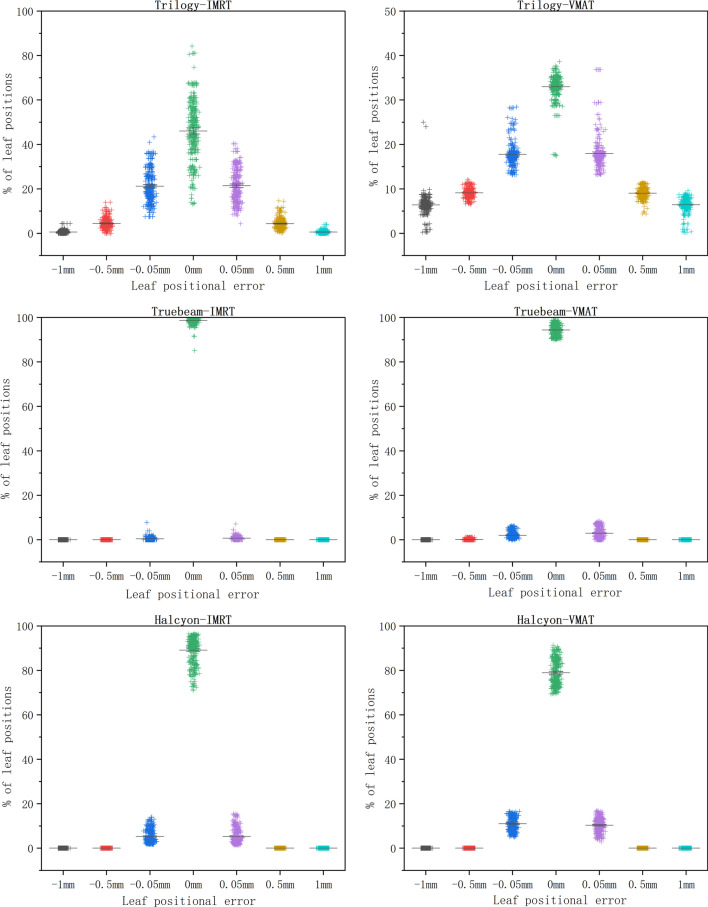
Percentages of different leaf positioning errors in the three accelerators with IMRT and VMAT techniques. The vertical coordinates represent percentages of leaf positioning errors. The horizontal coordinates represent the values of different leaf positioning errors

In TrueBeam, leaf position error above the threshold (1 mm) were not found in IMRT and VMAT plans. The specific percentages of different leaf position errors are shown in the subsequent analysis.

#### Halcyon

No correlation was observed between the maximum leaf RMS and 95th percentile error and the maximum leaf speed in IMRT and VMAT plans. The maximum leaf RMS error had no correlation with the mean leaf speed in IMRT and VMAT plans (R = 0.28, 0.34). The 95th percentile error was found to be correlated with the mean leaf speed (R = 0.60 and 0.50 in IMRT and VMAT, *p* < 0.05). Additional file 1: Fig. 5 shows the correlation between the 95th percentile error and mean leaf speed in IMRT and VMAT plans of Halcyon.

**Fig. 5:**
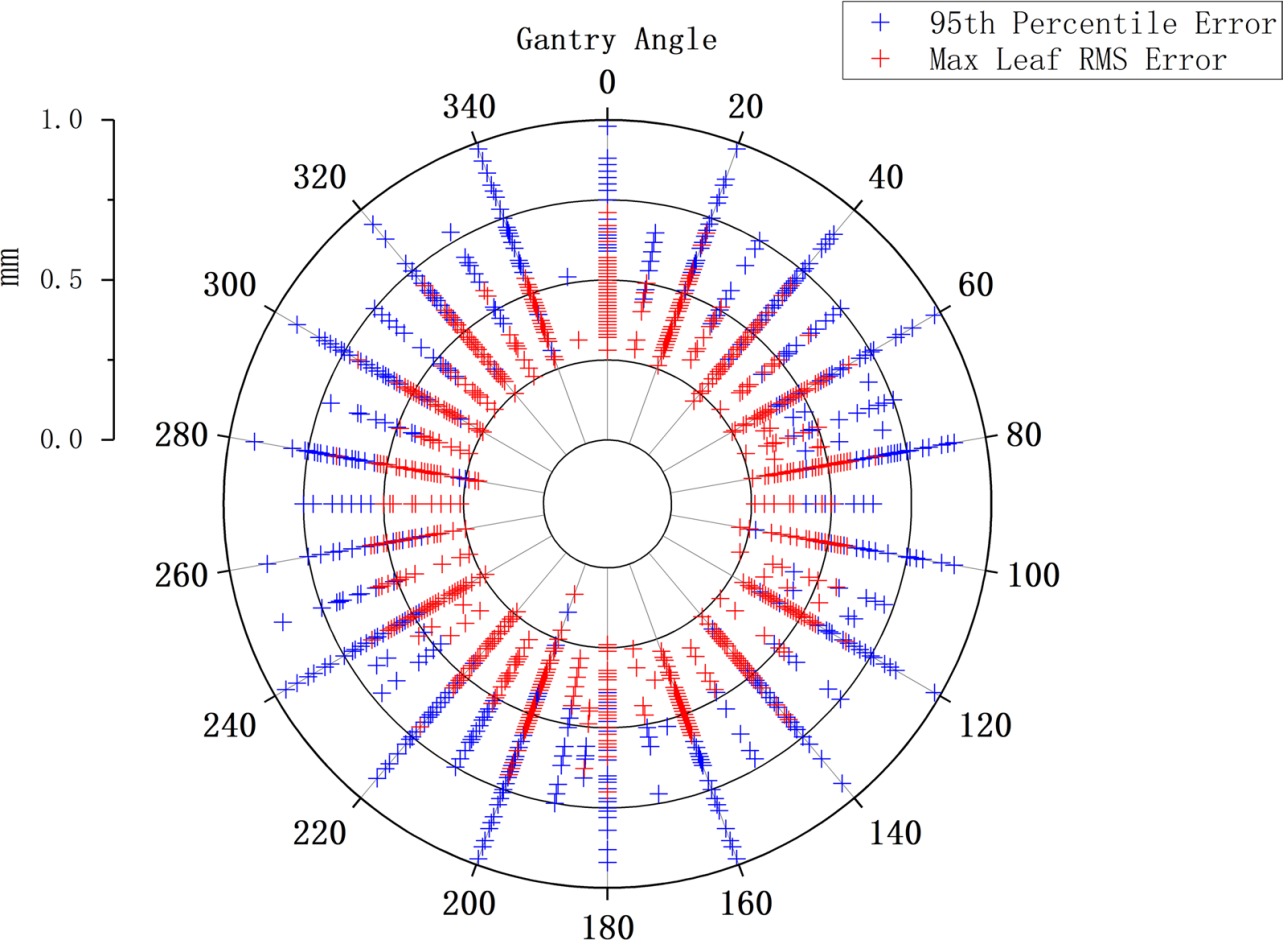
The radar plot of the maximum leaf RMS and 95th percentile error at different gantry angles in Trilogy with IMRT technique. Blue represents the 95th percentile error and red represents the maximum leaf RMS error

Like TrueBeam, leaf positioning error exceeding 1 mm was not found in IMRT and VMAT plans in Halcyon.

### Percentages of leaf positioning errors

The percentages of different leaf positioning errors are shown in Fig. 4. Almost all leaf positioning errors occured within ± 0.05 mm in TrueBeam. The data dispersion between 0 and ± 0.05 mm of the leaf positioning error in Halcyon was higher than that in TrueBeam, indicating that the leaf positioning accuracy of TrueBeam was better than that of Halcyon. The leaf positioning errors in VMAT were higher than those in IMRT for all accelerators.

### Correlation between leaf positioning error evaluation parameters and gantry or arc angles

In IMRT plans, no correlation was found between the two leaf-positioning-error evaluation parameters and the gantry angles, and the number of failed leaves was not correlated with the gantry angles as well. As shown in the radar plot in Fig. 5, this plot is composed of IMRT data from the Trilogy. No significant aggregation bias was seen for the maximum leaf RMS and 95th percentile error at different gantry angles, and angles with small values of the evaluation parameters do not necessarily represent small leaf errors, possibly due to the fact that these angles were set less in the treatment plan, as the number and angles of the fields in all plans were inconsistent. In TrueBeam and Halcyon, the data distributions of the two leaf-positioning-error evaluation parameters and the gantry angles were similar to that in Trilogy. Similar results were also generated between the degrees of arc and the evaluation parameters in the VMAT plans, showing that the leaf positioning errors of all three accelerators were independent of the gantry and arc angles for IMRT and VMAT techniques.

## Discussion

The study based on log files showed the variability in the 95th percentile error and the maximum leaf RMS error for all three accelerators, regardless of IMRT or VMAT techniques. In IMRT plans, the MLCs deviations of Trilogy accelerator were significantly higher than those of the other two machines, and is correlated with the mean and maximum speed of the MLCs. The MLC construction of Trilogy and TrueBeam are the same, and the maximum leaf speed is all set to 25 mm/s. The main reason for the MLC-positioning-error differences of the Trilogy and TrueBeam is that the active design of the MLC controller reduces the delay effect. Trilogy uses the passive MLC controller with the communication delay, but no communication delay is observed in TrueBeam because of the active MLC controller. Olasolo-Alonso et al. [16] have demonstrated that the leaf positioning errors caused by the MLC communication delay is significant and may be greater than that caused by factors such as friction, complexity and the gravity, since correcting for that, the leaf positioning errors and maximum RMS errors are weakly correlated with the mean and maximum speed of leaves. Their results agreed with ours. The differences in the percentages of different leaf positioning errors in Fig. 4 demonstrated that TrueBeam's active MLC controller does bring a great improvement compared to Trilogy's passive MLC controller, and the results are similar to previous studies [18]. In TrueBeam, the maximum RMS errors of the MLCs were not related to the maximum speed of the MLCs in both IMRT and VMAT plans. In VMAT plans of TrueBeam, both the maximum leaf RMS and the 95th percentile errors were correlated with the mean leaf speed, which were same as the results of Olasolo-Alonso et al. [16]. In Halcyon, we found that the two evaluation parameters of leaf positioning errors were only related to the mean speed of the MLCs. In both IMRT and VMAT plans, the mean values of maximum leaf RMS and 95th percentile errors of the Halcyon were slightly larger than those of the Truebeam, and were significantly lower than those of the Trilogy, as shown in Figs. 4 and Additional file 1: Fig. 1. The reason may be that although the Halcyon accelerator uses an active MLC controller, the maximum leaf speed was set to 50 mm/s and the dual-layer MLC is different from the other two accelerators. In Halcyon 1.0, the proximal leaves cannot shape the beam. Thus, when the proximal leaves are confined to the boundary of the primary collimator, the overlapping distal leaves are confined to the same position as the proximal leaves. The leaf-positioning-error evaluation parameters of Halcyon showed slightly worse than those of the Truebeam due to the differences in the leaf speed, the beam type, and MLC construction. We found that the maximum and mean leaf speed of Halcyon were higher than those of TrueBeam, which was the mainly reason why leaf-positioning-error evaluation parameters of Halcyon were slightly higher than those of the TrueBeam in both IMRT and VMAT techniques. FFF mode is used on Halcyon while only FF plans were analyzed on TrueBeam. The application of different technologies may also account for the different results between the two accelerators.

In the statistics of the mean and maximum speed of the MLCs, it was found that the maximum speed of some leaves exceeded the nominal maximum speed. The reason may be that the maximum leaf speed included the beam-hold state in this study. Another reason may be the non-compliance with the maximum leaf speed constraint in leaf sequencing algorithm. It was reported that 14% fields in sliding window IMRT technique exceeded the maximum speed limit in multiple leaves due to the sudden movement of bank B at the end of the MLC sequence [19].

In both IMRT and VMAT plans of the Trilogy, a strong correlation between the 95th percentile error and the number of failed leaves (threshold: 1 mm) was found in our study, as demonstrated in Additional file 1: Fig. 3. The number of failed leaves was not correlated with the gantry angles. In TrueBeam and Halcyon, leaf positioning error exceeding 1 mm was not found, and the 95th percentile error showed a correlation with the mean leaf speed. The maximum leaf RMS error was also correlated with the leaf speed in some of the data. Therefore, the leaf positioning error is mainly determined by the leaf speed, independent of the gantry angle, which is similar with the results of previous study [20].

The maximum leaf RMS and 95th percentile errors are very important as two parameters recommended by TG-142 and TG-198 to assess the leaf positioning errors. These parameters may reveal the characteristics of the leaf positioning error for different type of accelerators, which is helpful for understanding the accelerator performance and setting the thresholds of leaf positioning error. The setting of the action threshold for leaf positioning deviation is controversial. If the tolerance of leaf positioning deviation is set too high, it will lead to excessive deviation of leaf positioning and resulting in dose delivery error, while setting it too small may increase the number of delays, thus increasing the treatment time and dosimetric error. Some authors recommended an action threshold of 2 mm for dynamic MLC positioning deviation [14]. Hernandez et al. [19] suggested that the action threshold for MLC positioning deviation should be 1.5 mm. The action threshold of leaf positioning deviation is set to 1 mm in the Suncheck software we used in this study. In the Trilogy accelerator, a large number of leaf positioning errors exceeding the threshold were found, but none was found in TrueBeam or Halcyon. Therefore, we also believe that a 1 mm action threshold might be strict for Trilogy, and a 1.5 mm action threshold might be appropriate. However, based on the results in this study, tightening the action threshold of leaf positioning deviation to 1 mm is obviously more suitable for the Truebeam and Halcyon.

McGarry et al.  [21] concluded that Truebeam has higher MLC positioning accuracy than Varian Clinacs in VMAT delivery, but they did not recommend specific threshold standard. In addition, synchronous comparison of C-series, T-series, and O-series Varian accelerators for the evaluation parameters of leaf positioning errors have not been studied in the past. Our research shows that the two leaf-positioning-error evaluation parameters of TG142 and TG-198 should be properly tightened in different types of machines, instead of using the unified recommended value of 3.5 mm.

Although the VMAT technique of the three accelerators showed significantly higher leaf positioning deviations than IMRT due to factors such as gantry rotation and dose rate variation, there is no absolutely meaningful deviation component of the leaf positioning deviation for both techniques. As shown in Fig. 4, in Trilogy, the overall dispersion of VMAT data was greater than that of IMRT, and the data values cannot be compared to IMRT in a one-to-one correspondence. That is, there will be smaller values in the analysis parameters of VMAT and larger values in the analysis parameters of IMRT and the difference between IMRT and VMAT data values was very small, especially in the TrueBeam and Halcyon accelerators. One reason is that the cases studied in this research were not unified, and there will be differences in the complexity of different plans. The planning complexity results in highly modulated MLC motion, which increases the difficulty of executing dynamic leaves precisely in place. This study selected cases randomly. Although each accelerator contained plans with different complexity (more than five treatment sites), the correlation between the planning complexity parameters and the positioning accuracy of the leaves was not evaluated. The second reason is that the active MLC controller can better execute the treatment plan, regardless of IMRT or VMAT. Therefore, we do not recommend a stricter assessment threshold for IMRT with the dynamic sliding window technique than for VMAT in the study.

In this study, due to the different types of machines, the mechanical parameters (such as leaf speed, number and structure of leaves, dose rate, accelerator service time and FFF or FF mode) can't be completely unified. In addition, the difference in leaf width (for example, the central 5 mm width leaves are used more frequently than the 1 cm leaves on the outside), and the difference between treatment plans (plan complexity) may also be the influencing factors. A limitation of this study is that it does not include the treatment data of FFF plans on TrueBeam, and it may be one-sided for Truebeam. Therefore, it is pointed out that the results of this study are only for Truebeam with FF mode. The prescription doses of treatment plans analyzed in this study were between 1.8 Gy and 3 Gy per fraction. No plans with a higher dose per fraction were evaluated. In addition, the limited number of log files may not be an advantage, and a large amount of data may bring more inspiration, because the long-term operation of the accelerator may require more dynamic observation. Our future work is to increase the FFF plans on TrueBeam, and to link this recommendation threshold with the mechanical threshold of the accelerator, continue to expand the collection of log files containing more parameters, and then conduct more in-depth research.

In summary, for both IMRT and VMAT techniques, we recommend that different thresholds should be set according to the type of accelerator. In Trilogy, the thresholds of the 95th percentile error and the maximum RMS error are set to 1.5 mm and 1 mm, respectively. In TrueBeam and Halcyon accelerators, the thresholds of the 95th percentile error and the maximum RMS error are set to 1 mm and 0.5 mm, respectively.

These settings are established from log files when plans are delivering for different treatment sites and are suitable for dynamic IMRT and VMAT. The parameters enable treatment centers with the same equipment to evaluate MLC positioning deviation more quickly and detect MLC positioning deviation before reaching the action threshold, which further helps to understand the MLC control systems of different types of accelerators.

## Conclusions

The study demonstrates the variability of leaf positioning accuracy for different types of accelerators performing IMRT or VMAT, and uniform thresholds for evaluation parameters are inaccurate. The recommended action thresholds of MLC positioning deviations are 1.5 mm for Trilogy, and 1 mm for Truebeam and Halcyon, respectively. For the evaluation parameters recommended by TG-142 and TG-198, the 95th percentile error and the maximum RMS error are suggested to be tightened to 1.5 and 1 mm for the Trilogy accelerator respectively. In TrueBeam and Halcyon accelerators, 1 and 0.5 mm for the 95th percentile error and maximum RMS error, respectively, are considered appropriate.

## Supplementary Information

**Additional file 1.** Supplementary data.

## Data Availability

The data are available upon request.
