# Supplementary material for: Comparison of MLC positioning deviations using log files and establishment of specific assessment parameters for different accelerators with IMRT and VMAT
Source: Radiat Oncol. 2022 Jul 16;17:123. doi: 10.1186/s13014-022-02097-0 (PMC9288677; doi:10.1186/s13014-022-02097-0)

Supplementary data

Supplementary Table 1. Specific characteristic data for 420 treatment plans

Treatment site	Head&Neck		Chest		Pelvis		Breast		Others	
Planning mode	IMRT	VMAT	IMRT	VMAT	IMRT	VMAT	IMRT	VMAT	IMRT	VMAT
Corresponding number of plans	16*, 15×, 8#	19*, 21×, 11#	19*, 21×, 15#	9*, 8×, 9#	17*, 16×, 34#	25*, 24×, 37#	12*, 13×, 6#	9*, 9×, 3#	6*, 5×, 7#	8*, 8×, 10#
Total numbers of fields/arcs	135*, 128×, 73#	42*, 48×, 14#	102*, 112×, 82#	28*, 25×, 23#	129*, 120×, 246#	54*, 52×, 64#	87*, 101×, 45#	37*, 37×, 12#	28*, 23×, 33#	27*, 26×, 23#
Width of fields in X direction (cm)	15.15±2.83*	14.93±0.98*	9.21±2.60*	15.21±0.98*	16.07±3.26*	12.55±2.41*	10.50±3.22*	9.84±2.80*	10.90±1.46*	11.17±2.09*
	15.28±3.57×	15.29±0.46×	9.11±2.84×	14.19±2.38×	13.72±4.81×	15.56±4.86×	10.37±4.01×	10.26±2.35×	12.12±2.37×	11.82±2.41×
	15.23±1.93#	——#	9.33±2.43#	——#	14.89±3.03#	——#	9.67±2.70#	——#	11.40±2.06#	——#
Width of fields in Y direction (cm)	18.50±2.46*	19.68±3.95*	11.34±3.77*	19.28±4.41*	19.79±3.32*	22.29±6.51*	18.24±5.31*	17.43±4.30*	13.11±2.22*	11.94±2.45*
	17.47±3.61×	20.10±3.23×	11.75.2±5.35×	16.52±6.23×	20.17±8.31×	22.98±4.75×	15.35±6.84×	20.58±4.14×	15.68±2.48×	13.21±3.38×
	19.60±1.94#	——#	10.59±2.83#	——#	20.2±1.48#	——#	19.34±3.58#	——#	14.91±3.98#	——#
Fractional prescription dose (number of cases)	2.0Gy(30)<br>2.2Gy(9)	2.0Gy(40)<br>2.2Gy(11)	2.0Gy(31)<br>1.8Gy(24)	2.0Gy(20)<br>1.8Gy(6)	2.0Gy(37)<br>1.8Gy(30)	2.0Gy(53)<br>1.8Gy(33)	2.0Gy(31)	2.0Gy(21)	3.0Gy(3)<br>2.0Gy(15)	2.0Gy(24)<br>2.2Gy(2)

Notes: * means Trilogy; × means Truebeam; # means Halcyon; Halcyon has no JAW, the width of Field X/Y in IMRT plans of Halcyon were got by manual measurement of the maximum opening diameter of the Field MLC, and no manual measurement was made for VMAT plans

Supplementary Fig. 1 Comparisons of maximum leaf RMS errors and 95th percentile errors among the three accelerators with IMRT and VMAT technique

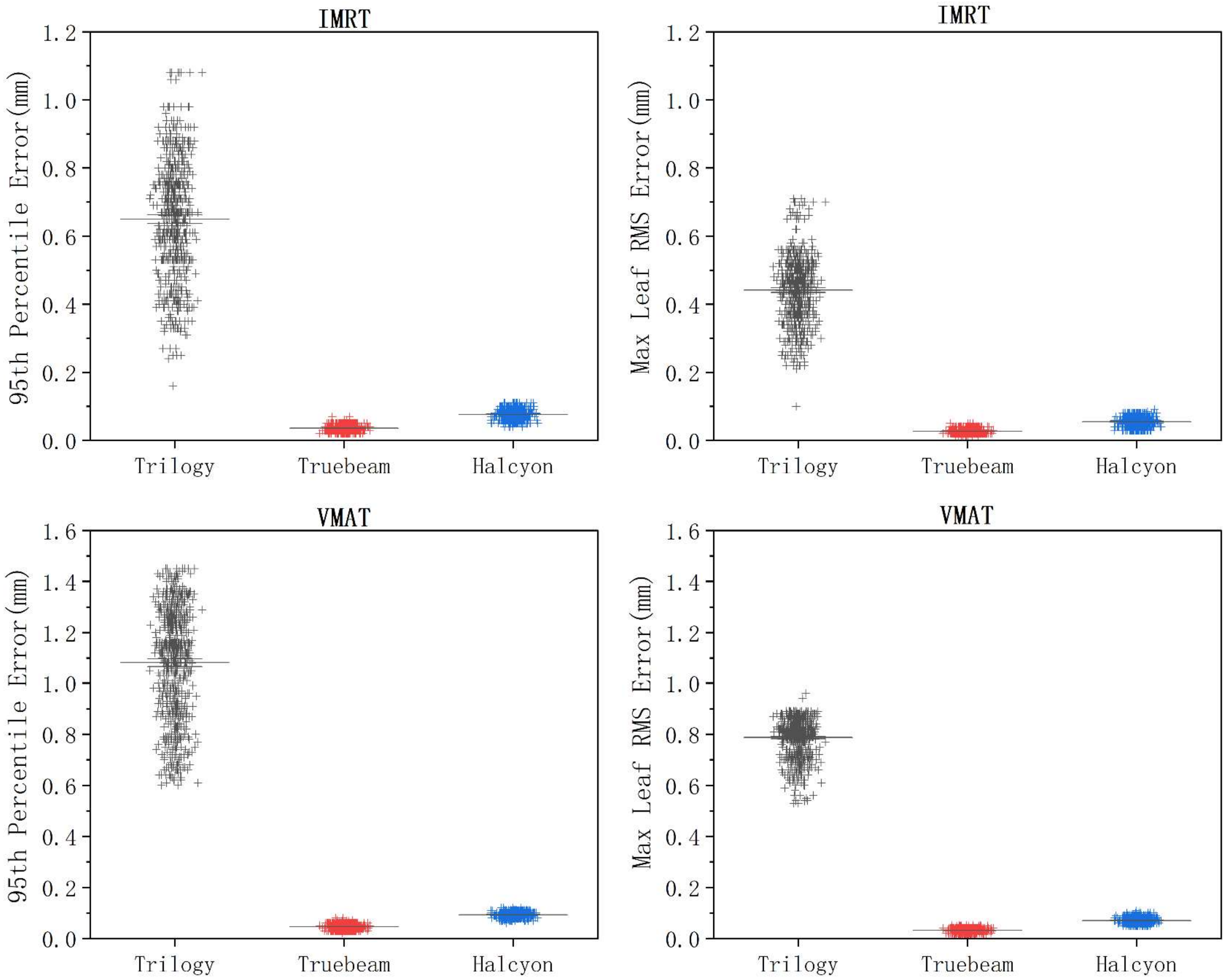

Supplementary Fig. 2 The density plots of correlation between the maximum leaf RMS and 95th percentile error and leaf speed in Trilogy with VMAT technique

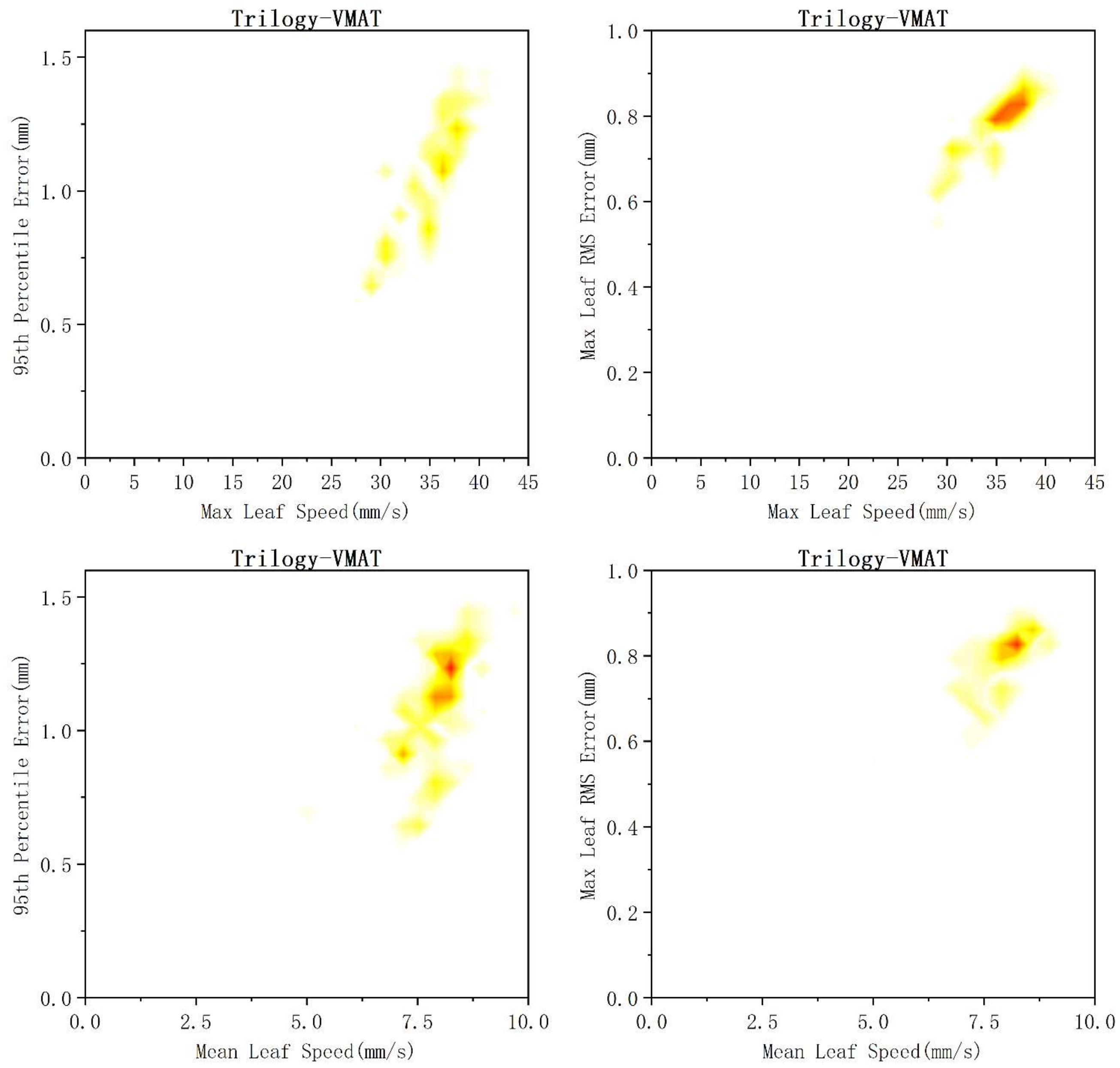

Supplementary Fig. 3 The density plots of correlation between the number of failed leaves and the 95th percentile error and mean leaf speed in Trilogy with IMRT and VMAT technique

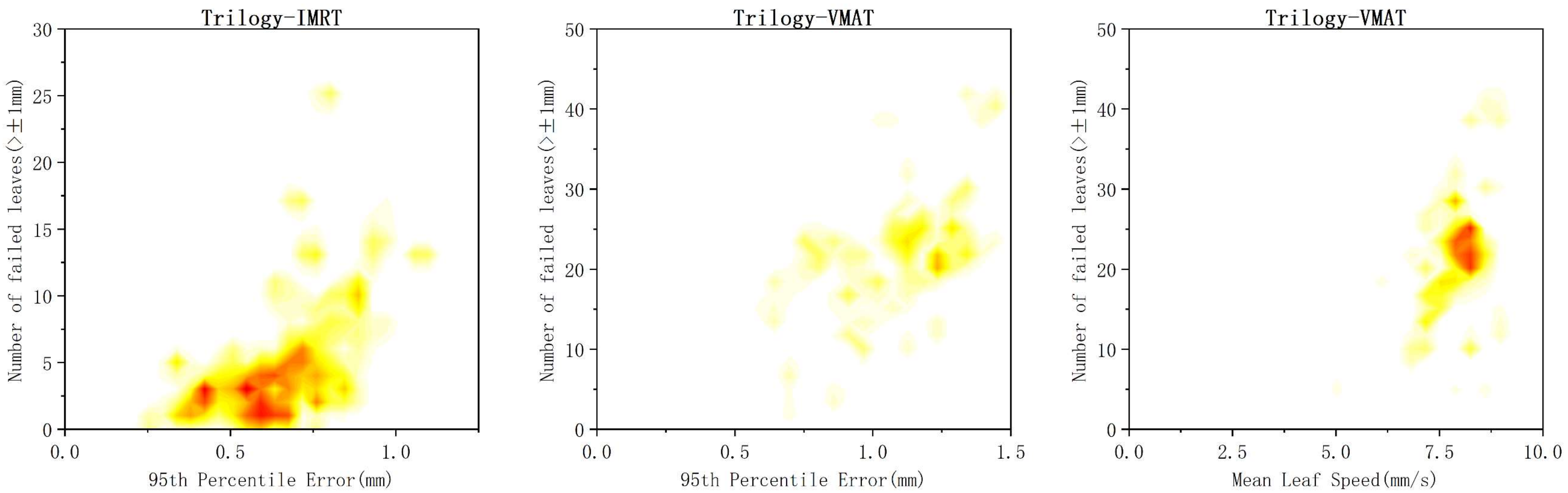

Supplementary Table 2. Correlation analysis between evaluation parameters

		Trilogy		Truebeam		Halcyon	
		IMRT	VMAT	IMRT	VMAT	IMRT	VMAT
Pearson	95th-RMS	0.59	0.86	0.61	0.70	0.61	0.58
	95th-MMS	0.29	0.84	0.52	0.27	0.36	0.39
	95th-AMS	0.67	0.61	0.60	0.70	0.60	0.50
	95th-FLN	0.55	0.55	*	*	*	*
	RMS-MMS	0.65	0.87	0.41	0.21	0.22	0.24
	RMS-AMS	0.70	0.72	0.47	0.57	0.28	0.34
	RMS-FLN	0.51	0.53	*	*	*	*
	MMS-AMS	0.29	0.66	0.60	0.21	0.41	0.36
	MMS-FLN	0.36	0.48	*	*	*	*
	AMS-FLN	0.37	0.55	*	*	*	*

Notes: 95th : 95th percentile errors; RMS: maximum leaf RMS errors; MMS:max MLC speed; AMS: average MLC speed; FLN: number of fail leaves

Supplementary Fig. 4 The density plots of correlation between the maximum leaf RMS and 95th percentile error and leaf speed in TrueBeam with IMRT and VMAT technique

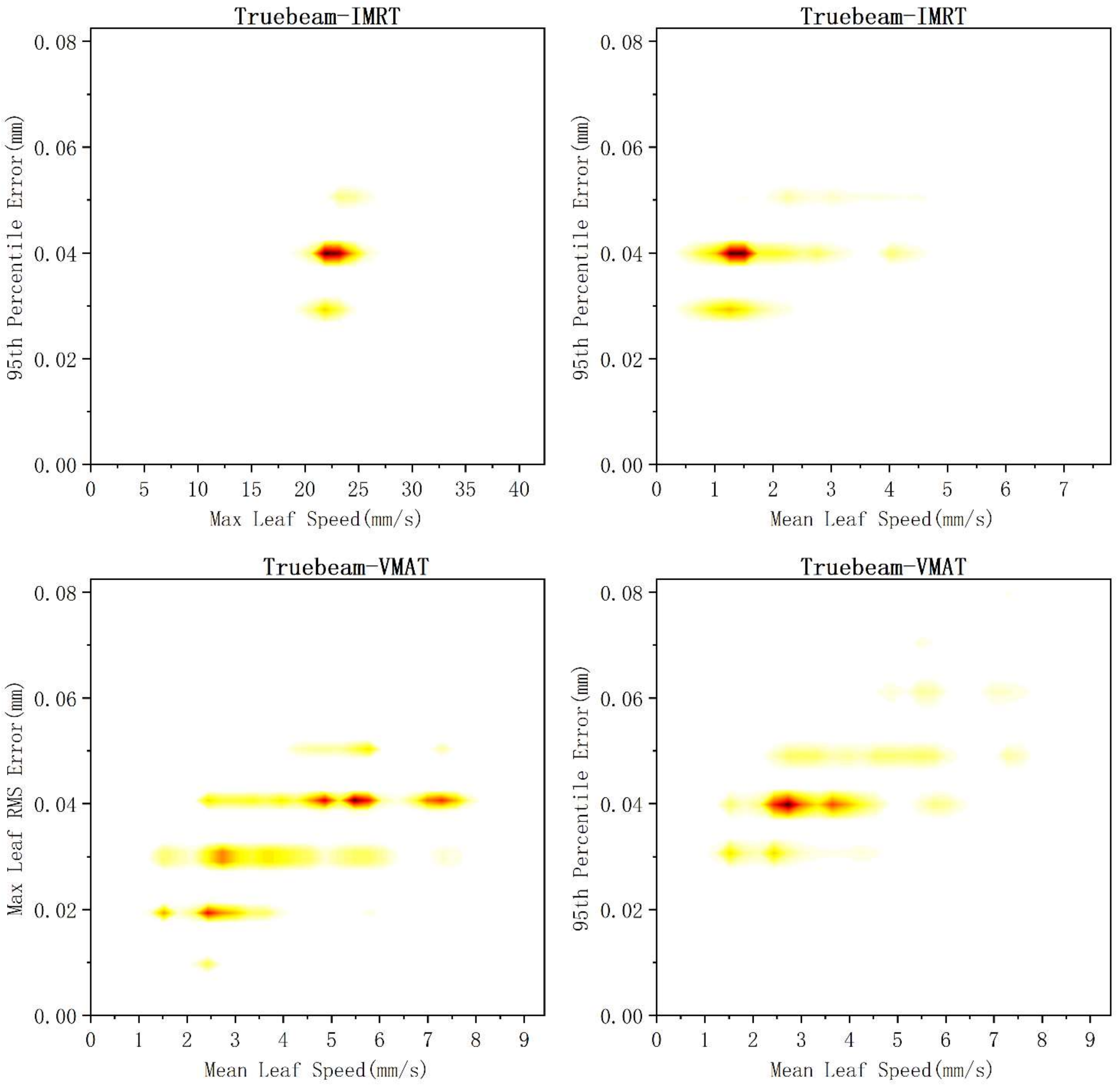

Supplementary Fig. 5 The density plots of the correlation between the 95th percentile error and mean leaf speed in IMRT and VMAT plans of Halcyon

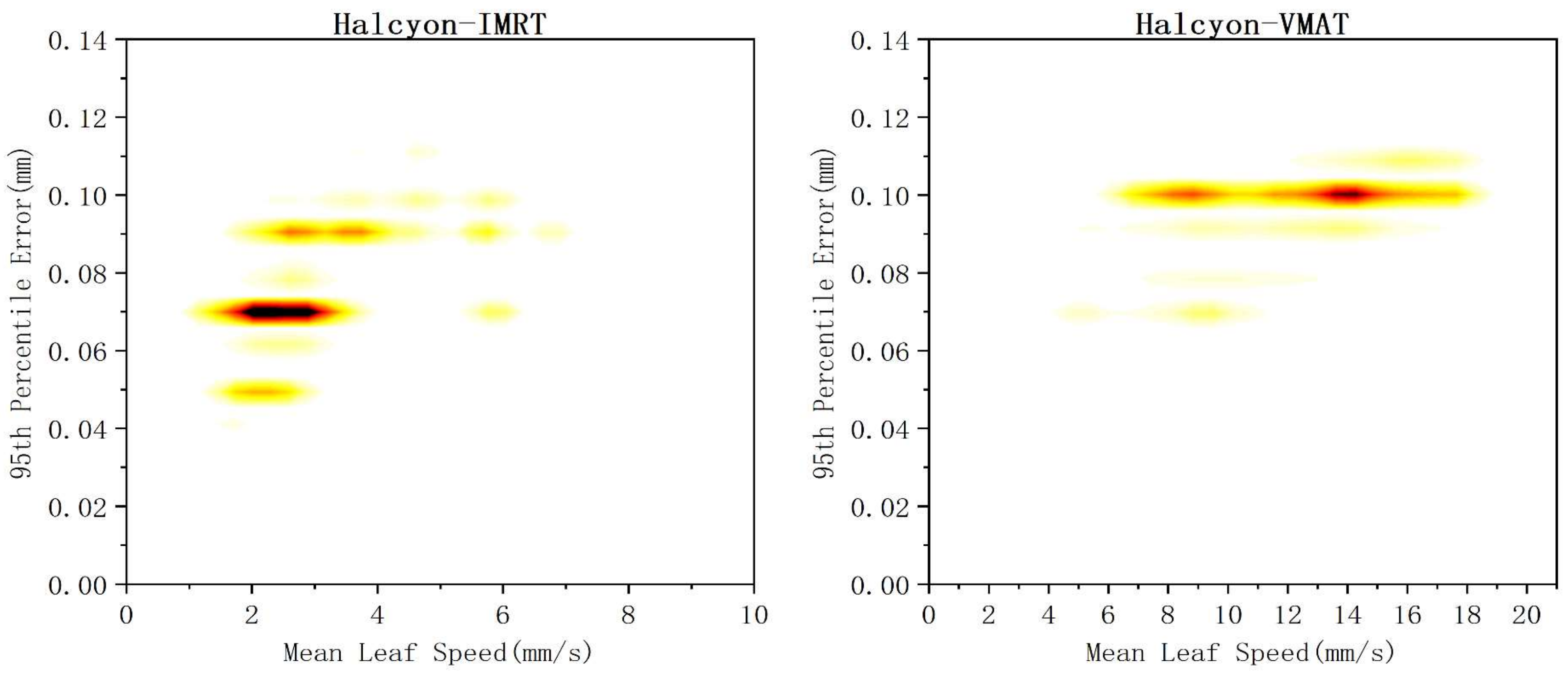

Supplement: Supplementary file 1 — Additional file 1. Supplementary data. [file 13014_2022_2097_MOESM1_ESM.pdf]
